# *Temnothorax rugatulus* ant colonies consistently vary in nest structure across time and context

**DOI:** 10.1371/journal.pone.0177598

**Published:** 2017-06-21

**Authors:** Nicholas DiRienzo, Anna Dornhaus

**Affiliations:** University of Arizona, Department of Ecology and Evolutionary Biology, Tucson, AZ, United States of America; University of Sheffield, UNITED KINGDOM

## Abstract

A host of animals build architectural constructions. Such constructions frequently vary with environmental and individual/colony conditions, and their architecture directly influences behavior and fitness. The nests of ant colonies drive and enable many of their collective behaviors, and as such are part of their ‘extended phenotype’. Since ant colonies have been recently shown to differ in behavior and life history strategy, we ask whether colonies differ in another trait: the architecture of the constructions they create. We allowed *Temnothorax rugatulus* rock ants, who create nests by building walls within narrow rock gaps, to repeatedly build nest walls in a fixed crevice but under two environmental conditions. We find that colonies consistently differ in their architecture across environments and over nest building events. Colony identity explained 12–40% of the variation in nest architecture, while colony properties and environmental conditions explained 5–20%, as indicated by the condition and marginal R^2^ values. When their nest boxes were covered, which produced higher humidity and lower airflow, colonies built thicker, longer, and heavier walls. Colonies also built more robust walls when they had more brood, suggesting a protective function of wall thickness. This is, to our knowledge, the first study to explicitly investigate the repeatability of nestbuilding behavior in a controlled environment. Our results suggest that colonies may face tradeoffs, perhaps between factors such as active vs. passive nest defense, and that selection may act on individual construction rules as a mechanisms to mediate colony-level behavior.

## Introduction

Many animals build architectural constructions, from nests, to hives, to webs, and dams, and research has examined their construction rules, function or plasticity [1–8]. Construction activities are often responsive to environmental and organism condition: for example, honeybees alter comb construction in response to different seasonal demands [2], and black widow spiders modify their nest structure in response to body condition [6]. Yet, one aspect of animal architecture that has not been considered, especially in social insects, is whether individual colonies consistently differ in the constructions they create (but see [6, 9] for non-social examples). In other words, do some colonies repeatedly build nests with fundamentally different architectural features than others (e.g. thicker walls, larger chambers, etc.), even after accounting for colony demographics and environmental variables? Such differences may drive fitness tradeoffs by altering individual or colony ability to defend, forage, and respond to the environment.

Consistent differences among individuals in behavior, which are also known as animal ‘personalities’, are widespread: in many species, certain individuals or colonies are more aggressive, active, or bold than others, affecting foraging, dispersal, sexual selection, and a host of other fitness-related processes [10, 11]. Architectural constructions mediate many of these same processes, and thus consistent individual- or colony-level differences in architecture may also impact different aspects of organism fitness. Social insects are an ideal model for such studies as they virtually all construct nests [3]. Indeed, several studies have demonstrated that ant colonies consistently differ in behavior [11, 12], and that this may be mediated in part by nest architecture [13–15]. Yet, these inferences are from field-based studies, and the observed differences in architecture may be a byproduct of environmental effects driving structural differences [13, 16]. Thus, it may be that ants are building different structures to alter worker behavior, or that the environment restricts architecture which in turn impacts behavior. While the direction of causality is hard to establish, the potential evolutionary consequences may nonetheless be significant: if colonies purposefully build structures with specific architecture in order to express a certain behavioral phenotype (e.g. aggressive), then selection will act on the behaviors underlying the building process, not necessarily aggressive behavior itself. On the other hand, if environmental conditions affect what structures are built, then building behavior may not be subject to selection, and instead aggressive behavior may be exposed to selection.

Here, we use *Temnothorax rugatulus* rock ants to investigate two main questions. First, how do colony traits, such as worker or brood number, and environmental conditions influence nest architecture? Second, do colonies demonstrate consistent differences in nest architecture over time and across different environmental contexts? Ants of the genus *Temnothorax* have long been a model for studying nest building. They build simple, two dimensional nests, often in flat cavities between rocks [17]. They are additive builders, enclosing existing cavities with substrate, rather than subtractive builders who excavate soil to create nests [17]. *Temnothorax* ants prefer larger nest cavities and narrow entrances [18], perhaps to limit moisture loss or access by competitors/predators. Furthermore, *Temnothorax spp*. will readily move if a superior nest location is discovered [18], indicating the strong preference for these features and likely fitness benefits associated with them.

## Materials and methods

All colonies (n = 18) were collected in the Catalina Mountains (32°13'44.1"N, 110°57'19.2"W) near Tucson, AZ in March, 2016. *T*. *rugatulus* are not a protected species, and collection permits were not required given they were collected in the Coronado National Forest. After collection the colonies were brought into the laboratory and given a plastic container (17.5cm x 12.5 x 6), a crevice to build in made from two glass slides (102mm x 76) separated by a strip of 1.5mm thick cardboard at the rear along with a small piece at the front to keep the slides from collapsing, and *ad lib* food and water (See S1 Fig for a photo of the experimental setup). Colonies were maintained under these conditions for two weeks before beginning the nestbuilding assays.

Nestbuilding trials began by placing each colony in a new nestbuilding container. Each container contained a water vial, a 1ml vial of sucrose solution, and 10 frozen drosophila. Opposite the glass slide we placed approximately 4g of store bought ceramic rocks (average size = 1.72mm, average weight = 4.87mg) for the ants to use as building substrate. To start a trial, the top slide of the colony's former nest was removed, and the ants and brood were gently transferred to the new nestbuilding arena using a fine paintbrush. All colonies were allowed to build for 12 days. Each nest was then photographed for later analysis. After the 12 day span, the colony was transferred to a new nestbuilding arena, forcing them to build another new nests. Prior to transfer we removed all nestbuilding substrate from the container in order to calculate the total substrate used. We manipulated environmental conditions by either covering the arena with a fitting lid, left slightly ajar to allow for oxygen exchange, or leaving it uncovered, a condition that primarily reduces humidity and increases airflow. Although ambient humidity in the laboratory fluctuated (between 0–25%), we found that humidity inside was increased when the colony was covered. Tests showed that one hour after transfer laboratory humidity was 21%, while the container humidity was 32%, and differences remained for a seven day period (4 hours, laboratory humidity = 16%, container humidity = 23%; 1 day, laboratory = 16%, container = 22%, 7 days, laboratory = 16%, container = 21%) (VWR digital temperature/humidity monitor, model 35519–048). All colonies built a total of four nests (two in covered, two in uncovered conditions). Colonies were assigned to either building in the uncovered or covered condition first.

**Fig 1:**
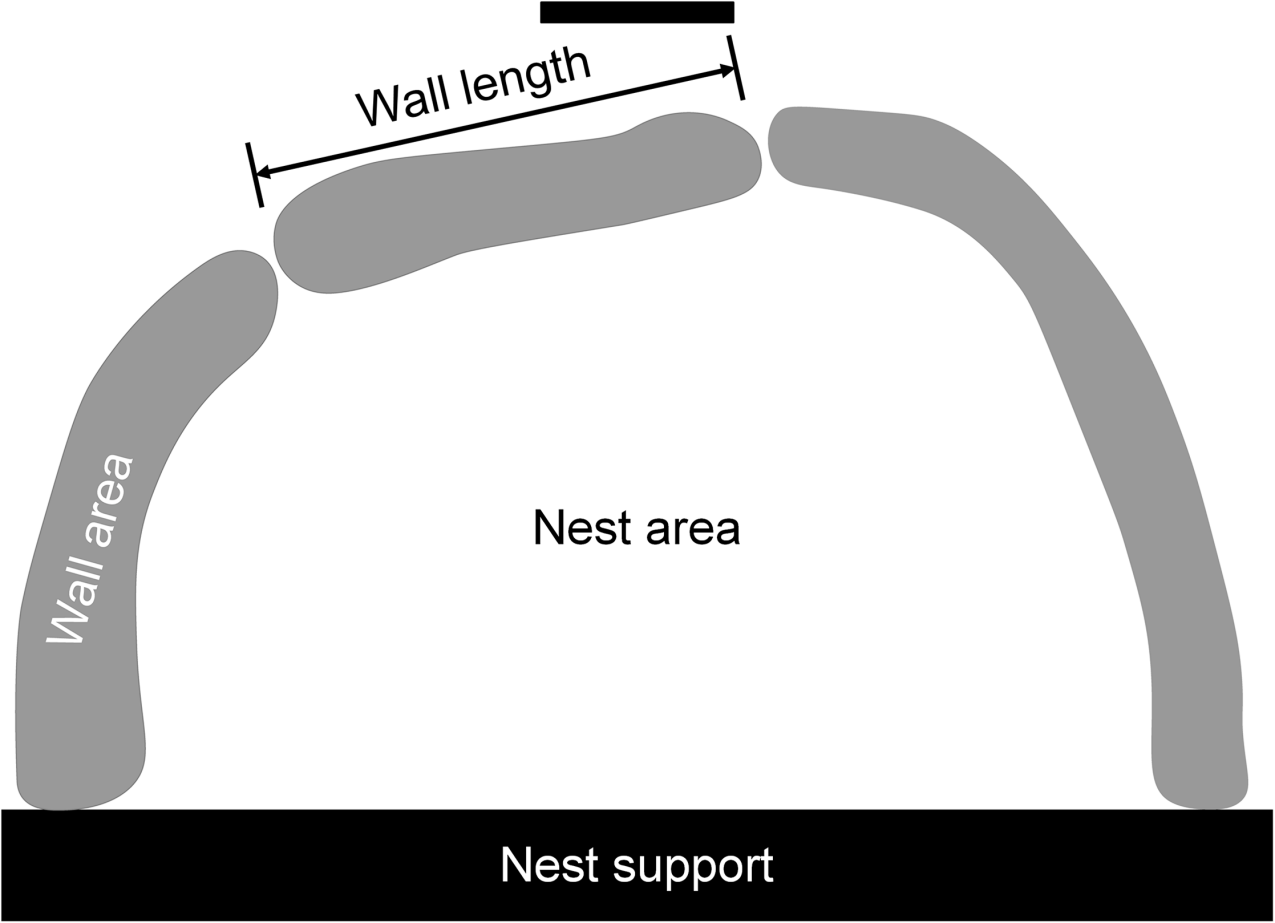
Top-down diagram of *Temnothorax* nest structure. Wall length and area were measured as the sum of the measures from each segment.

We used the software ImageJ to measure, from photographs of the nest, the area of the wall built, the length of the wall built, and the internal nest area (Fig 1). We also counted the number of workers (9–81) and brood (22–395) from the photographs.

## Statistical analysis

We assessed differences in the various aspects of nest structure using linear mixed models. Our response variables were wall area, wall length, material used, and internal area. Fixed effects were the number of brood, number of workers, trial number, and if the arena was covered. Linear mixed models determined that trial order (covered or uncovered first) had no influence on any response variables (wall area, p = 0.350; wall length, p = 0.563; material used, p = 0.457; internal area, p = 0.996), and thus was not included as a fixed effect. Continuous variables were centered to a mean of zero and scaled to a standard deviation of 1. Colony ID was included as a random effect. All response variables were modeled with Gaussian distributions. To determine which of these main effects best predicted each response, we used an information theoretic approach. We made models with all possible combinations of the main effects (See Tables A-D in S1 File), and fit them with the R package lme4 [19]. We used the Akaike Information Criterion with small sample size correction (AICc) for model comparison. We calculated the delta AIC as the difference in AIC between the top model and the next best fit model. Models within two delta AIC were deemed to be statistically indistinguishable [20]. We calculated the AIC weights, which describe the probability of the model being the best fit relative to the other models in the set. We calculated the amount of variation explained by the fixed effects alone (marginal R^2^), and the amount of variation explained by both fixed and random effects (conditional R^2^) for the top models.

We calculated the adjusted colony-level repeatabilities as the amount of variance associated with colony ID over the sum of colony ID variance and residual variance. All variance components were taken from the fully parameterized models (i.e. including all measured main effects). We calculated 95% confidence intervals though parametric bootstrapping procedures (number of simulations = 5000). Parametric bootstrapping is a procedure that repeatedly generates data from the estimated model and subsequently refits model to the generated data. Confidence intervals can then be measured from the distribution of refitted model parameters. We tested for significance of the repeatability values through likelihood ratio tests comparing the full model to one with the individual random effect removed [21].

## Results

**Fig 2:**
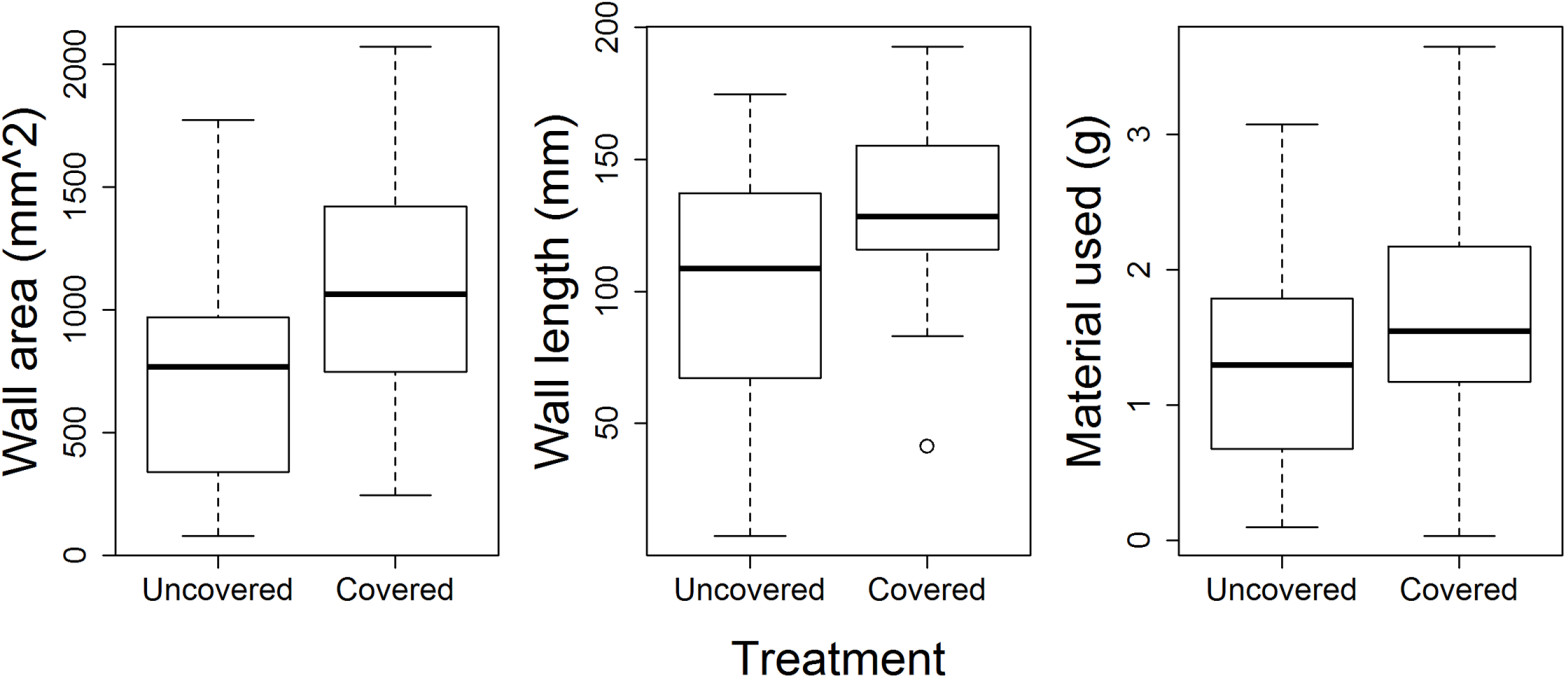
Boxplots illustrating the effect of the nestbox being covered on wall area, length, and the amount of material used. All differences were significant (area p <0.001, length p <0.001, material used p = 0.014)

**Fig 3:**
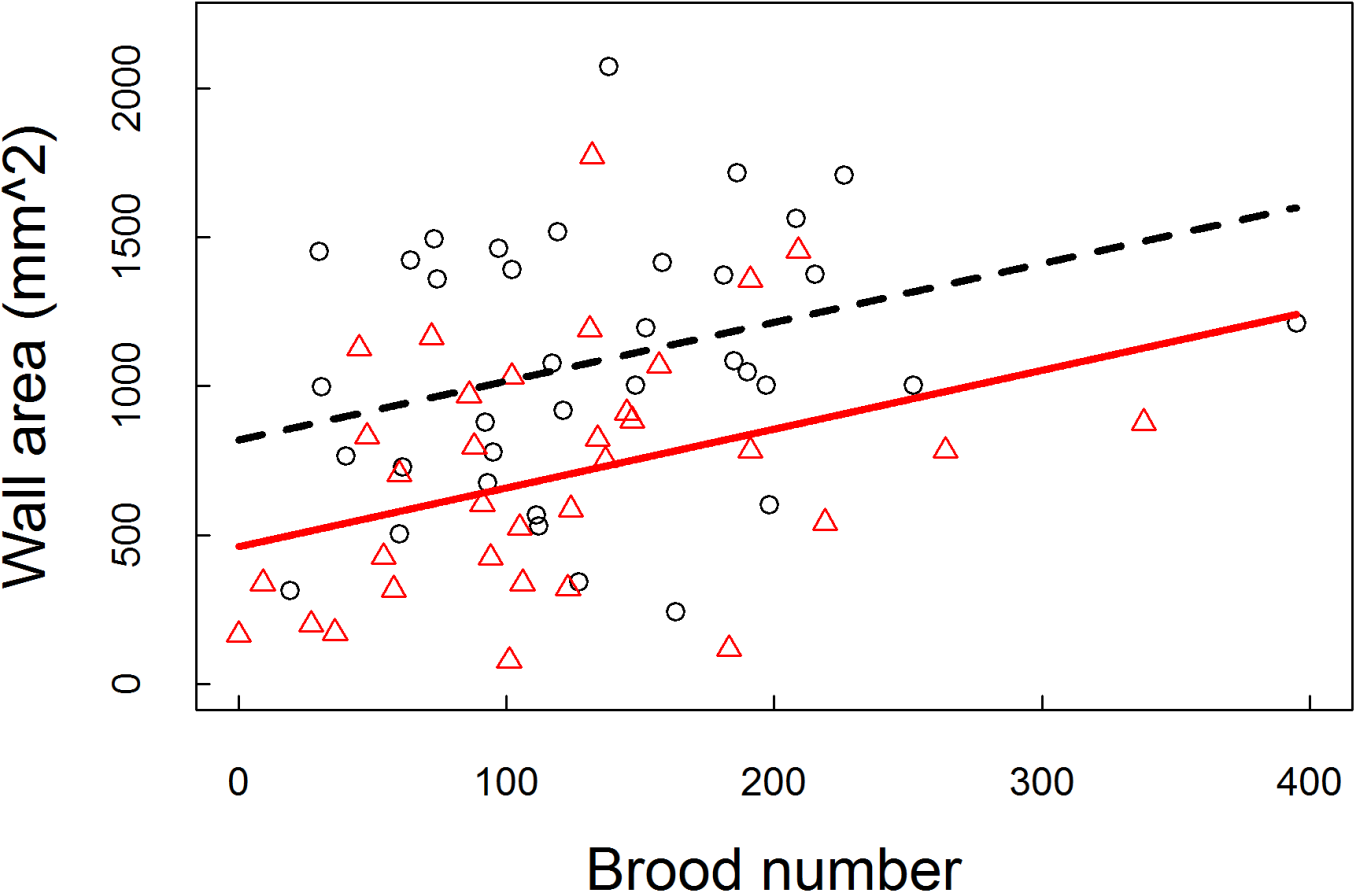
The relationship between wall area and brood number. Black circles and dashed line indicate covered observations and the associated relationship, while red triangles and solid line indicates the uncovered observations and associated relationship.

Model comparison indicated that although the fully parameterized model was overall the best fit for most nest measures (See Tables A-D in S1 File for AIC results), generally the only universally important predictor within the models was environmental condition (whether the nest box was covered). When colonies were covered they built a greater wall area (ß = 358.279 ± 75.606, p < 0.001), with longer walls (ß = 31.095 ± 8.676, p < 0.001), and used more material (ß = 0.339 ± 0.138, p = 0.014), but the same internal nest area (ß = -200.803 ± 133.341, p = 0.132) (Fig 2). The number of brood had only an effect on wall area, such that colonies with more brood built thicker walls (ß = 146.253 ± 66.441, p = 0.001, Fig 3). The number of workers did not affect any of the measured nest parameters (Table 1).

**Table 1:** Top linear mixed model outputs predicting nest properties as indicated by AIC model comparison. Table of best fit linear mixed models.

	Wall area				Wall length				Substrate used				Nest area			
Random effects	Estimate				Estimate				Estimate				Estimate			
Colony ID	98208				223.081				0.249				143249			
Residual	92331				1225.894				0.331				288300			
Fixed Effects	ß	SE	z	p	ß	SE	z	p	ß	SE	z	p	ß	SE	z	p
Intercept	656.761	132.310	4.964	<0.001	83.564	12.978	6.439	<0.001	1.257	0.154	8.165	<0.001	4622.007	212.401	21.761	<0.001
Brood number	146.253	66.411	2.202	0.028	9.350	6.051	1.545	0.122	-	-	-	-	-5.119	105.854	-0.048	0.961
Worker number	-89.944	71.108	-1.265	0.206	0.891	6.055	0.147	0.883	-	-	-	-	197.238	109.404	1.803	0.071
Nest number	21.784	36.484	0.597	0.550	7.443	4.079	1.825	0.068	-	-	-	-	-98.243	63.523	-1.547	0.122
Covered	358.279	75.606	4.739	<0.001	31.095	8.676	3.584	<0.001	0.339	0.138	2.455	0.014	-200.803	133.341	-1.506	0.132
Marginal R2	0.210				0.192				0.048				0.114			
Conditional R2	0.617				0.316				0.457				0.408			

Estimates for brood and worker number are both based on the scaled and centered values. A total 70 nests were built by the 18 colonies.

We found that colony ID predicted 12–40% of the variance in nest structure (Table 1). Furthermore, colonies generally showed consistent differences across the different trials and environmental conditions, as indicated by the significant levels of repeatability in the wall area (r = 0.515, 95% CI = 0.237–0.738, LRT = 14.454, p < 0.001), material used (r = 0.479, 95% CI = 0.190–0.712, LRT = 12.544, p < 0.001), and internal area (r = 0.332, 95% CI = 0.060–0.590, LRT = 7.552, p = 0.002), although not for the wall length (r = 0.153, 95% CI = 0.000–0.436, LRT = 1.118, p = 0.092). These are moderate to high levels of repeatability as a meta analysis (Bell 2009) found that the average repeatability of 759 estimates taken from 114 studies was 0.37 [22]

## Discussion

**Fig 4:**
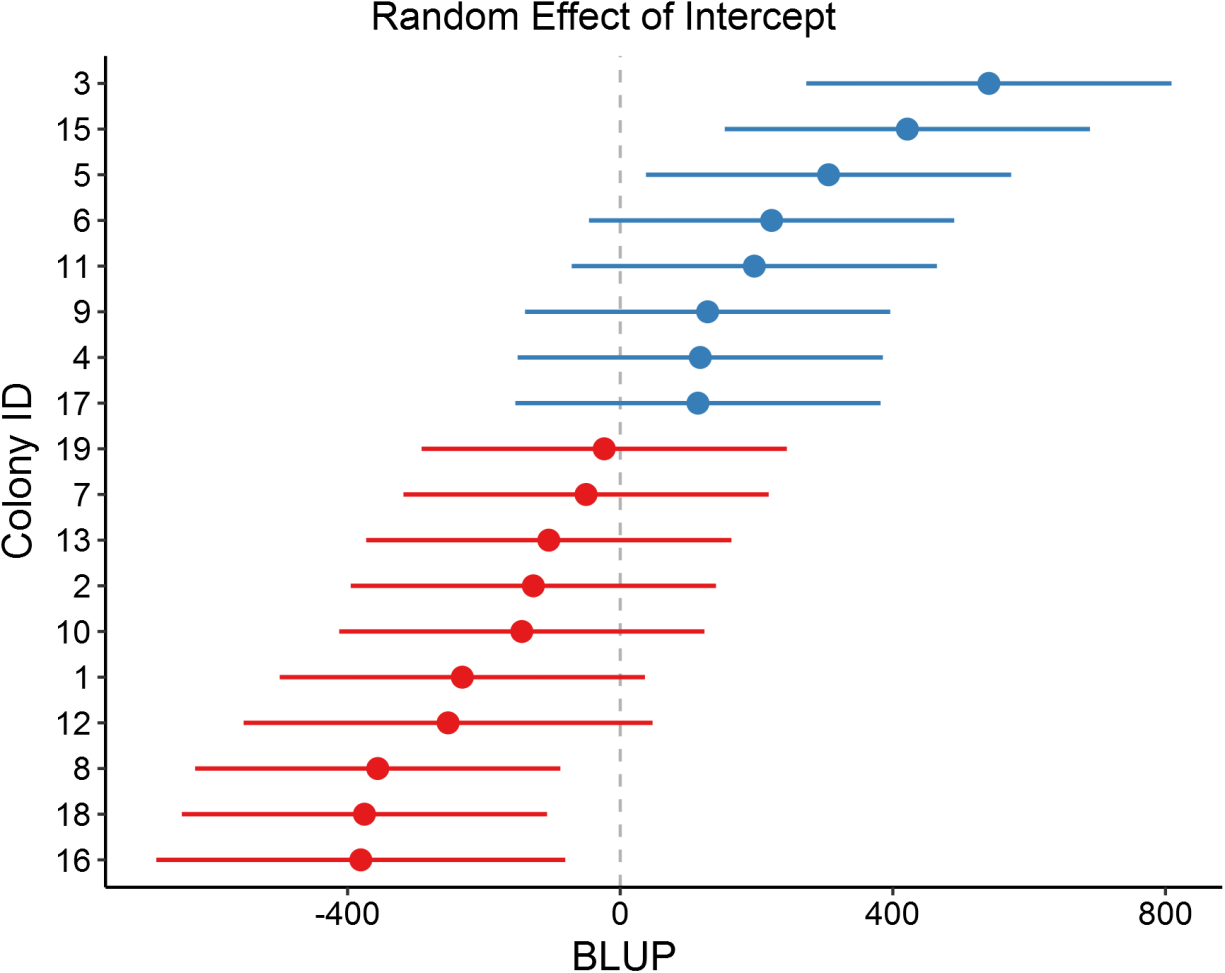
Colony variation in intercept as estimated by the linear mixed model predicting wall area. BLUP (best linear unbiased predictor) illustrates the colony estimated deviation from the LMM intercept estimate for wall area. Values below zero indicate a colony consistently built nests with a lower wall area than the estimated intercept, while values above zero indicate they consistently built walls with a larger area.

We found significant among-colony variation, as indicated by the high levels of repeatability, in several of the architectural parameters, but not others. In particular, some repeatedly build thicker, more robust walls (Fig 4), while others build thinner walls, but colonies did not differ in the length of wall built. It is worth noting that the fixed size of the crevice provided by the glass slides may have limited the expression of wall building: colonies may not have been able to express differences in wall length, as the maximum length was the perimeter of the slide. Colonies responded to being covered, and thus the increase in humidity and reduction of airflow, by increasing nest structure investment (e.g. thicker, longer walls). Furthermore, colony traits such as worker and brood number, and environmental conditions (covered vs. uncovered) together only explained 5–20% of the variation in nest structure, while the addition of colony ID explained an additional 12–40%. This indicates that much of nest architecture is determined not by external environmental factors or colony size, but instead by some intrinsic factor of the colony such as genotype, neural or hormonal differences, or differences in experience.

Collectively, these results highlight that there are extensive among-colony differences in nest structure and building behavior, while colonies respond in a similar manner to environmental variation. Given the costs associated with desiccation, colonies may invest more in nest structure when humidity is high and/or airflow is low [13, 23], as was likely the case when we covered the arena. Furthermore, colonies build thicker walls when they have more brood. This highlights that overall fuller, more robust walls may be a mechanism to deal with the external environment and/or protection, but colonies vary in their willingness to invest in them, as indicated by the among-colony differences in wall area (Fig 4). One possible explanation is that colonies vary in their defensive strategies: Some colonies may invest more in passive defense mechanisms (e.g. wall thickness), while others invest more in active defense mechanism (e.g. more workers that will defend against intruders) [16]. It is important to note that despite the significant response to the container being covered (Fig 2), we do not know which environmental variable drove the variation. Future studies will directly investigate these questions by looking at how worker placement (inside/outside nest) and colony response varies with nest structure and specific environmental conditions.

The colony differences demonstrated here may have a genetic underpinning or be the result of plasticity in response to environmental conditions; e.g. colonies may have experienced subtle microhabitat differences in the field, and this may carry over to the observed differences. Overall, this study highlights that nest architecture is a part of colony-level personality. This topic has recently come into focus as colonies have been shown to consistently differ in several behavioral tendencies, including foraging, aggression, and activity [16, 24–26]. Yet, a remaining question is how colony composition may drive these architectural differences: are there more highly productive builders, or do all individuals display a greater tendency to build [27]? This study also adds social insects to the growing body of literature demonstrating within-species variation in construction architecture [6, 28–30]. Given that these nest structures mediate certain aspects of behavior [13, 15, 31], there may be significant evolutionary consequences of this variation. Specifically, several behaviors often considered to represent aspects of colony performance, such as foraging and defense [16, 25, 26], may not be under direct selection. Instead, selection may act on the construction rules which result in structures that facilitate the appropriate behavioral expression for the colony.

## Supporting information

S1 File Tables of AIC rankings. File containing AIC rankings of models predicting total wall area, lenght, material used, and internal area.
(DOCX)

S2 File Experimental data. CSV file containing the data collected during this experiment.
(CSV)

S1 Fig Photograph of nestbuilding setup used in this experiment. The plastic box measures 17.5cm x 12.5 x 6, while the glass slides measure 102mm x 76. They are separated by a 1.5mm thick piece of cardboard.
(TIF)
