# Supplementary material for: Temnothorax rugatulus ant colonies consistently vary in nest structure across time and context
Source: PLoS One. 2017 Jun 21;12(6):e0177598. doi: 10.1371/journal.pone.0177598 (PMC5479500; doi:10.1371/journal.pone.0177598)
Supplement: S1 File — File containing AIC rankings of models predicting total wall area, lenght, material used, and internal area. (DOCX) [file pone.0177598.s001.docx]

| Model | Structure | AICc | dAICc | Df | weight |
| --- | --- | --- | --- | --- | --- |
| 16 | brood + worker + nest + covered | 988.9 | 0.0 | 7 | 0.957 |
| 13 | Brood + worker + covered | 995.8 | 6.9 | 6 | 0.031 |
| 14 | Brood + nest + covered | 998.0 | 9.1 | 6 | 0.010 |
| 15 | Worker + nest + covered | 1001.3 | 12.5 | 6 | 0.002 |
| 8 | Brood + covered | 1004.9 | 16.0 | 5 | <0.001 |
| 10 | Worker + covered | 1007.8 | 19.0 | 5 | <0.001 |
| 11 | Nest + covered | 1009.3 | 20.4 | 5 | <0.001 |
| 12 | Brood + worker + nest | 1015.5 | 26.6 | 6 | <0.001 |
| 5 | covered | 1015.9 | 27.1 | 4 | <0.001 |
| 6 | Brood + worker | 1022.5 | 33.6 | 5 | <0.001 |
| 7 | Brood + nest | 1024.2 | 35.3 | 5 | <0.001 |
| 9 | Worker + nest | 1028.8 | 39.9 | 5 | <0.001 |
| 2 | Brood | 1031.3 | 42.5 | 4 | <0.001 |
| 4 | Nest | 1036.8 | 47.9 | 4 | <0.001 |
| 3 | Worker | 1036.8 | 48.0 | 4 | <0.001 |
| 1 | Null | 1045.0 | 56.1 | 3 | <0.001 |

Table A: Rankings of models predicting total wall area as determined by AICc.

| Model | Structure | AICc | dAICc | Df | weight |
| --- | --- | --- | --- | --- | --- |
| 16 | Brood + worker + nest + covered | 690.5 | 0.0 | 7 | 0.714 |
| 14 | Brood + nest + covered | 693.4 | 3.0 | 6 | 0.163 |
| 15 | Worker + nest + covered | 695.8 | 5.3 | 6 | 0.050 |
| 13 | Brood + worker + covered | 695.9 | 5.4 | 6 | 0.047 |
| 8 | Brood + covered | 699.1 | 8.6 | 5 | 0.010 |
| 11 | Nest + covered | 699.4 | 9.0 | 5 | 0.008 |
| 10 | Worker + covered | 700.0 | 9.5 | 5 | 0.006 |
| 5 | Covered | 703.7 | 13.2 | 4 | <0.001 |
| 12 | Brood + worker + nest | 705.7 | 15.2 | 6 | <0.001 |
| 7 | Brood + nest | 708.8 | 18.3 | 5 | <0.001 |
| 6 | Brood + worker | 709.1 | 18.6 | 5 | <0.001 |
| 9 | Worker + nest | 711.7 | 21.2 | 5 | <0.001 |
| 2 | Brood | 712.3 | 21.9 | 4 | <0.001 |
| 3 | Worker | 714.4 | 23.9 | 4 | <0.001 |
| 4 | Nest | 716.0 | 25.5 | 4 | <0.001 |
| 1 | Null | 718.7 | 28.2 | 5 | <0.001 |

Table B: Rankings of models predicting total wall length as determined by AICc.

| Model | Structure | AICc | dAICc | Df | weight |
| --- | --- | --- | --- | --- | --- |
| 5 | covered | 156.8 | 0.0 | 4 | 0.371 |
| 1 | null | 158.2 | 1.4 | 3 | 0.188 |
| 2 | brood | 159.1 | 2.2 | 4 | 0.121 |
| 8 | Brood + coverd | 159.1 | 2.3 | 5 | 0.112 |
| 4 | nest | 161.3 | 4.5 | 4 | 0.040 |
| 11 | Nest + covered | 161.4 | 4.5 | 5 | 0.038 |
| 10 | Worker + covered | 161.5 | 4.7 | 5 | 0.036 |
| 6 | Brood + worker | 162.6 | 5.7 | 5 | 0.021 |
| 13 | Brood + worker + covered | 162.8 | 5.9 | 6 | 0.019 |
| 3 | worker | 162.9 | 6.0 | 4 | 0.018 |
| 7 | Brood + nest | 163.8 | 7.0 | 5 | 0.011 |
| 14 | Brood + nest + covered | 164.6 | 7.7 | 6 | 0.008 |
| 9 | Worker + nest | 166.0 | 9.1 | 5 | 0.004 |
| 15 | Worker + nest + covered | 166.1 | 9.2 | 6 | 0.004 |
| 12 | Brood + worker + nest | 167.6 | 10.8 | 6 | 0.002 |
| 16 | Brood + worker + nest + covered | 168.4 | 11.6 | 7 | 0.001 |

Table C: Rankings of models predicting total material used as determined by AICc.

| Model | Structure | AICc | dAICc | Df | weight |
| --- | --- | --- | --- | --- | --- |
| 16 | Brood + worker + nest + covered | 1053.9 | 0.0 | 7 | 0.975 |
| 15 | Worker + nest + covered | 1062.6 | 8.7 | 6 | 0.013 |
| 13 | Brood + worker + covered | 1063.9 | 10.0 | 6 | 0.007 |
| 12 | Brood + worker + nest | 1065.3 | 11.4 | 6 | 0.003 |
| 14 | Brood + nest + covered | 1065.8 | 11.9 | 6 | 0.003 |
| 10 | Worker + covered | 1072.8 | 18.9 | 5 | <0.001 |
| 9 | Worker + nest | 1074.1 | 20.2 | 5 | <0.001 |
| 6 | Brood + worker | 1074.6 | 20.7 | 5 | <0.001 |
| 11 | Nest + covered | 1075.0 | 21.1 | 5 | <0.001 |
| 8 | Brood + covered | 1075.3 | 21.4 | 5 | <0.001 |
| 7 | Brood + nest | 1077.6 | 23.7 | 5 | <0.001 |
| 3 | Worker | 1083.4 | 29.4 | 4 | <0.001 |
| 5 | Covered | 1085.4 | 31.5 | 4 | <0.001 |
| 2 | Brood | 1086.4 | 32.5 | 4 | <0.001 |
| 4 | Nest | 1086.6 | 32.7 | 4 | <0.001 |
| 1 | Null | 1096.1 | 42.2 | 3 | <0.001 |

Table D: Rankings of models predicting total internal nest area as determined by AICc.
