# Supplementary figures and images for: Temnothorax rugatulus ant colonies consistently vary in nest structure across time and context
Source: PLoS One. 2017 Jun 21;12(6):e0177598. doi: 10.1371/journal.pone.0177598 (PMC5479500; doi:10.1371/journal.pone.0177598)

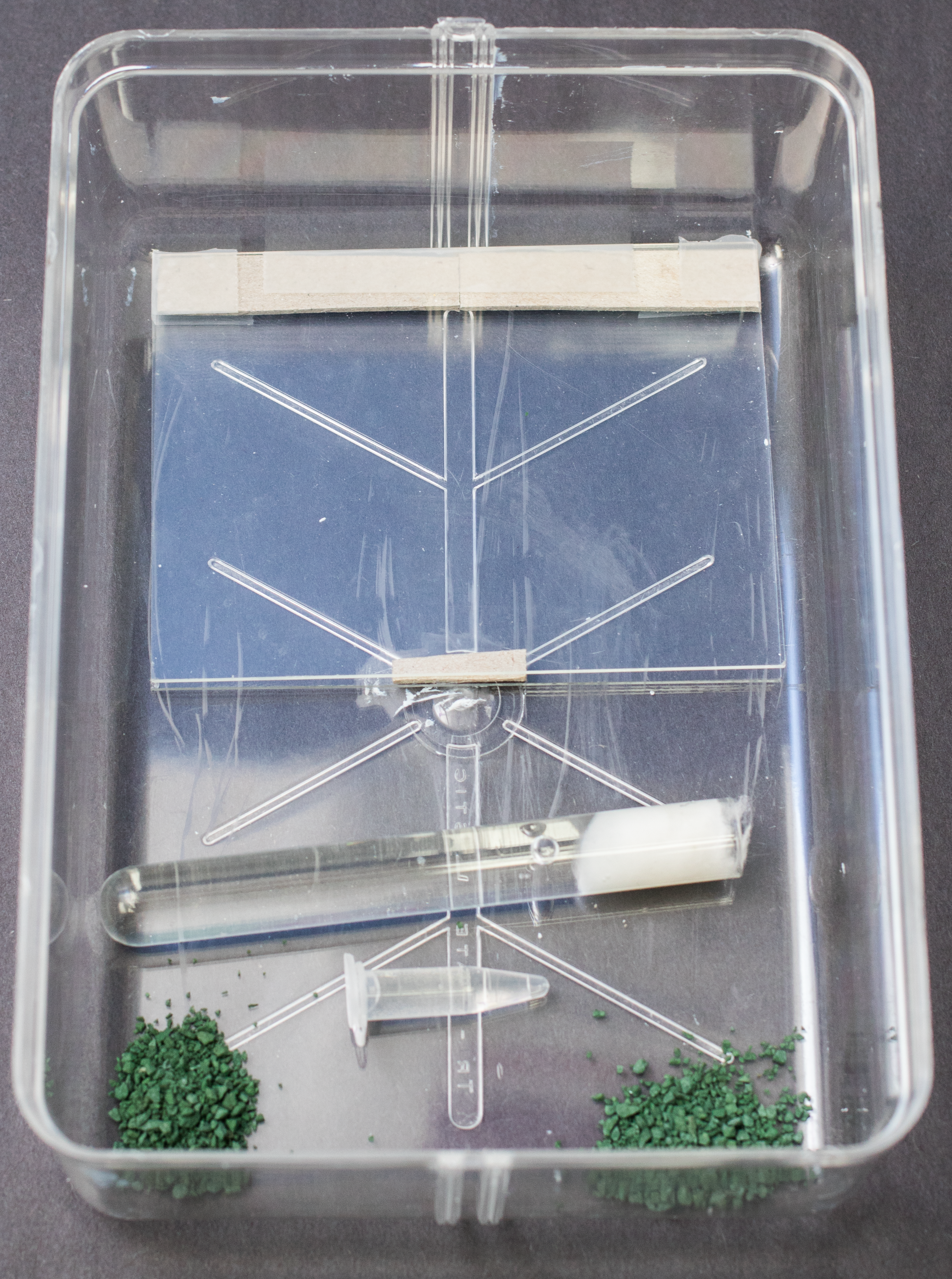

Supplement: S1 Fig — The plastic box measures 17.5cm x 12.5 x 6, while the glass slides measure 102mm x 76. They are separated by a 1.5mm thick piece of cardboard. (TIF) [file pone.0177598.s003.tif]
